# Frontal theta-gamma ratio is a sensitive index of concussion history in athletes on tasks of visuo-motor control

**DOI:** 10.1038/s41598-019-54054-9

**Published:** 2019-11-26

**Authors:** Dmitri Poltavski, Kyle Bernhardt, Christopher Mark, David Biberdorf

**Affiliations:** 1 Department of Psychology, 501 N Columbia Rd, Stop 8380, University of North Dakota, Grand Forks, 58202-8380 ND USA; 2 Department of Psychology, Salem State University, 352 Lafayette St., Salem, MA 01970 USA; 3 Valley Vision Clinic, 2200 S. Washington St., Grand Forks, 58201 ND USA

**Keywords:** Pattern vision, Human behaviour, Brain injuries

## Abstract

Patients with mTBI often show deficits in executive function and changes in neural activity. Similar changes in those with a history of mTBI (i.e. concussion), however, have not been consistently reported. Frontal theta-to-gamma frequency ratio has shown promise in EEG research in predicting performance on working memory tasks. In the present study we explored the sensitivity of the frontal theta-to-gamma relative power spectral density (PSD) ratio to the history of concussion in 81 youth athletes (18 with a history of concussion, ages 13–18) during the tests of the Nike Sensory Training Station that vary in working memory and processing speed demands and motor output requirements. The results showed that the theta-to-gamma relative PSD ratio was significantly lower in the concussion history group on the tests of target capture, perception span and hand reaction time. A principle component analysis further indicated that this metric reflects an underlying dimension shared by several visuo-motor control tests of the Nike battery. The results suggested persistent deficits in psychomotor ability in the athletes with a history of concussion that may have implications for diagnosis, rehabilitation and athletic training.

## Introduction

### Visual Skills in Sports and mTBI

The annual prevalence of sport-related concussion, according to the Centers for Disease Control and Prevention (CDC) estimate, is anywhere between 1.6–3.8 million in the U.S. alone^1^, which represents 5 to 9% of all sports-related injuries^2,3^. Concussions are the most common form of mild traumatic brain injury accounting for up 86% of all traumatic brain injury cases. Athletes typically return to play within a two-week period, as most concussions (80–90%) are believed to resolve within a 7–10 day period^4^. The risk of a repeat concussion, however, is 2–5.8 times higher in individuals with a history of a previous concussion^5,6^, which can lead to significant morbidity (e.g., 5-fold greater likelihood of mild cognitive impairment^7^).

Some of the most commonly observed persistent deficits following mTBI include deficits in visual processing speeds^8^, compromised working memory function^9^, difficulty concentrating and sustaining attentional focus on a target^10,11^, poor decision-making^12^ and reduced stress regulation^13^. All of these skills also appear critical to peak athletic performance^14,15^.

Specialized sports vision training (SVT) programs now routinely use integrated visual assessment systems, such as the Nike SPARQ Sensory Training Station, which can test and train a wide range of visual, sensorimotor and information processing skills that previous studies identified as important for sports^16,17^. The results of these studies strongly suggest that such visuomotor skills as dynamic visual acuity, depth perception, near-far quickness, perception span, eye-hand coordination, hand reaction time, and stimulus discrimination are directly related to athletic performance, and that enhancing these skills through specialized training may lead to better athletic and health outcomes.

Despite the station’s utility in assessment and training of perceptual and visual-motor skills important for athletic performance and mTBI, a recent study that directly compared a large sample of youth and college-level athletes with and without a history of concussion failed to detect any significant performance differences on all but one measure of the Nike Sensory Station (i.e. depth perception)^18^. These results are not necessarily surprising, as computerized neuropsychological assessments have not shown particular sensitivity to the history of mTBI^19–21^.

## EEG, Working Memory and mTBI

Neural measures can detect subtle changes in cognitive states not evident with changes in task performance^22,23^. Within EEG literature working memory has been investigated within the context of theta-gamma coupling both in the hippocampus and the cortex. Kaminski, Brzezicka, and Wrobel^24^ found that the theta/gamma cycle length ratio obtained from electrode Fz (frontal midline) significantly predicted performance on the digit span task, with greater ratios corresponding to better scores. These results provided further evidence to the theory originally proposed by Lisman and Idiart^25^ that within working memory individual memories are activated in different gamma cycles by a specific firing pattern of cells phase- locked to a theta cycle that is thought to organize memory storage. Each gamma wave representing a memory is locked to a specific phase within the theta cycle, which ensures the correct order of memory retrieval. Memory capacity is delimited by the number of phase-locked gamma cycles within the theta cycle.

Patients with TBI have demonstrated pronounced deficits in working memory paradigms, including the n-back task^26^, a form of memory task requiring prefrontal executive function. Imaging studies also suggest that mTBI patients may have fewer neural reserves available to them during working memory tasks as they do not increase brain activation patterns to the same extent as the non-mTBI controls at higher working memory loads^27,28^.

Working memory tasks, such as the n-back task are thought to represent a more general set of psychomotor tasks. Chaiken *et al*.^29^ defined these tasks as those that involve a significant perceptual and response load with a defining feature of a required complex perceptual discrimination or a production of a complex motor response. Through path analysis the researchers demonstrated that the psychomotor ability seems to be fairly unitary in nature and that tasks of visuo-motor control largely depend on the working memory capacity and temporal processing speeds.

Using an exploratory factor analysis the Applebaum group reduced the nine perceptual measures of the Nike Sensory Station to 3 distinct and interpretable dimensions: visual motor control, visual sensitivity and eye-quickness^30^. The Nike SST tasks of visuo-motor control (e.g. eye-hand coordination, Go/No-Go, hand response time and perception span) combine significant working memory demands and temporal processing speeds that determine timely and accurate motor output. Performance on these tasks in individuals with a history of mTBI could, thus, be investigated using EEG-based approaches that could potentially prove more sensitive to working memory demands than the behavioral measures of accuracy and speed.

## Current Study

While a very promising approach, phase detection and theta-gamma cross-frequency coupling (CFC) continues to present a technical and methodological challenge^31^ that may not be easily and practically resolved with turn-key commercial wireless EEG systems in the context of sensorimotor assessment in athletes. At the same time reductions in both global and frontal theta and gamma power have been reported in mTBI^32,33^.

The purpose of the present study was thus to explore the relationship between frontal theta and gamma power in the context of perceptual and visuo-motor assessment of youth athletes with and without concussion history.

We hypothesized that there would be significant differences between athletes with and without the history of concussion in the ratio of the frontal theta- to- gamma power during completion of visuo-motor tasks of the Nike Sensory Station, with lower ratios indicating reduced visual working memory efficiency and associated with poorer performance on these measures.

## Method

### Participants

Eighty-one youth participants (70 males and 11 females, ages 13–18) were recruited from local and public school extra-curricular athletic programs in ice-hockey via their respective activities directors and athletic program directors. Participants were recruited as part of a larger study intended to compare the effectiveness of two sports vision programs on oculomotor, sensory-motor, cognitive, and neurophysiological outcomes in youth hockey players. The study was conducted following the experimental protocol approved by the Institutional Review Board (IRB) of the University of North Dakota. The data were collected in accordance with the guidelines and regulation established by the protocol. The participation was voluntary, and the participants had the right to withdraw any time from the study.

Prior to enrollment into the study, informed consent was obtained from both parents/ legal guardians of youth between 13–18 as well as participating minors. Participants under 16 were also given a simplified assent form, in which the study and their participation was explained in plain every-day terms.

All enrolled participants underwent a standard optometric exam administered by a licensed optometrist on the premises of a local optometric clinic. Exclusionary criteria included strabismus, uncorrected astigmatism and anisometropia, as well as significant ocular pathology (excluding color deficiencies). As part of the optometric evaluation procedure refractive error and/or residual error for each eye under non-cyclopleged conditions were recorded and, if needed, a participant received soft sport contact lenses before any type of testing to correct visual acuity to at least logMAR of 0 (i.e. 20/20 on the Snellen chart) or better.

While youth athletes with a history of concussion were encouraged to participate in the study, those with a recent concussion (less than 12 weeks old) were also excluded. Concussion history was verified via completion of the ThinkFirst Concussion Quetionnaire^34^ and during a brief intake interview available as part of the Nike SST assessment. Eighteen participants reported having had at least one lifetime concussion (range 1–4, mean 1.83). Eight of these youth athletes reported having had their most recent concussion in the past year, while the remaining ten indicated having had a concussion over a year before their participation in the study. None of the participants with a history of concussion reported any lasting symptoms and all of them resumed their regular athletic and academic activities following their most recent concussion.

### Instruments

#### Nike sensory performance system

A detailed description of the Nike SPARQ Sensory Performance System (Nike SST) is provided in our earlier paper^16^. Here we reiterate the overall description of the system’s tests. The Nike SST is a computer-based vision assessment station that evaluates athletes on 9 sport-relevant visual and sensory performance skills. It consists of a single computer controlling two high-resolution liquid crystal display monitors (both 0.2 mm dot pitch): one 22-inch diagonal display and one 42-inch diagonal touch-sensitive display. Custom software controls the displays, input acquisition, and test procedures based on subject responses. Five of the tests are performed 16 feet (4.9 m) from the 22-inch display screen. The subject uses a handheld Apple iPod touch (Apple Corporation, Cuptertino, California), which is connected via wireless input to the computer so that it could interact with the station’s screen monitor. These tests include Visual Clarity, Contrast Sensitivity, Depth Perception (Stereopsis) at Far, Near-Far Quickness and Target Capture (dynamic visual acuity). The other 4 tests are performed with the subject positioned within arm’s length of the 42-inch touch sensitive screen mounted with the center of the screen at about eye-level. These tests include Perception Span, Eye-Hand Coordination (Peripheral Eye-hand response), Go/No Go and Hand Reaction Time (central eye-hand reaction and response time). Reliability and validity information of the Nike SST output parameters can be found in Erickson *et al*.^35^.

#### EEG

In the present study we utilized the B-Alert X10 device (ABM, Carlsbad, California) for wireless EEG recording along 9 channels that collect continuous electroencephalographic signals from electrode locations in the frontal (Fz, F3 and F4), central (Cz, C3 and C4) and parietal-occipital (POz, P3, and P4) areas. These electrode locations are predetermined by distances between sensors on a 9-sensor strip, the size of which varies depending on the distance between the subject’s nasion and inion. As per manufacturer’s recommendation, the medium strip was used with nasion-inion distances exceeding 34.5 cm and the small strip was applied when the distance was between 32.0 and 34.5 cm. Using these guidelines, sensor locations correspond to the International 10–20 system of scalp electrode placement. Disposable self-adhesive sponge electrodes filled with 0.4–0.6 cc of Synapse® conductive electrode cream were attached to the sensor strip before the headset’s placement on the participant’s scalp. Two mastoid leads with disposable electrodes served as reference and were placed over the participant’s mastoid bones on each side of the head.

The B-Alert X-10 has a 256 Hz sampling rate and sends radio signals using a 2.4 to 2.48 GHz radio transmitter to the B-Alert X-10 Live acquisition software that can be run from any PC using a USB receiver. Prior to signal transmission the unit also performs analog-to-digital conversion, encoding, and formatting.

### Procedure

All testing was conducted on the premises of a local optometric clinic. All participants provided informed consent upon their first visit to the clinic and before the optometric examination. All Nike SST and EEG testing sessions were completed after the optometric evaluation and on a separate day. Upon arrival to the lab, each participant was fitted with the EEG sensor strip that was plugged into the B-Alert X-10 wireless sensor headset. Once all channel impedance values were below the manufacturer recommended 40 kΩ, each participant first underwent a 15-minute neuropsychological evaluation consisting of three 5-minute computerized tasks programmed by the manufacturer of the acquisition software (ABM) to automatically generate cognitive state metrics (these statistics were not utilized in this study).

Following completion of the benchmark tests, participants underwent the Nike SST assessment. A total of nine Nike SST tests were completed by each participant. These tests included measures of static visual acuity, contrast sensitivity, depth perception, near-far quickness, dynamic visual acuity, perception span, eye-hand coordination, “Go/No-Go”, and hand reaction time. Each test was preceded by a brief practice session that included several trials. The measures could not be counterbalanced between participants, as the Nike SST software did not allow the administration order of the measures to be altered. Within the B-Alert X-10 Live Acquisition software, the continuous EEG stream was broken into intervals corresponding to the beginning and end of each Nike SST task. This was accomplished by manually placing start and end markers into the EEG file online during completion of the sensorimotor tasks (practice sessions were not included). Once the participant completed all the tasks, acquisition of EEG signals was terminated, the headset and the sensor strip were removed, the participant was debriefed and dismissed. Each assessment session lasted on average 60–65 minutes.

### EEG data acquisition and processing

Data were sampled and processed at 256 Hz using a band pass filter from 0.5–65 Hz (3 dB attenuation). Notch filters at 50, 60, 100, and 120 Hz were used to specifically target the removal of environmental noise (i.e., electrical interference) not fully attenuated by the band pass filter. The EEG signal was referenced to the linked mastoid-placed electrodes using the average voltage between the reference electrodes. The acquisition software^36^ used ABM algorithms to detect and remove artifacts (spikes, excursions, amplifier saturations, electromyography, and eye blinks) prior to power spectral density (PSD) computations. PSD was automatically computed by the B-Alert Live software using a 50% overlapping window across three, one-second data overlays (256 decontaminated data points each) and applying the Fast Fourier Transformation with Kaiser windowing for data smoothing. If more than 128 zero values were inserted for an overlay, the overlay was excluded from the epoch average; if 2 of the 3 overlays were rejected, the epoch was classified ‘invalid’ excluded from analysis.

This resulted in PSD values for each one-second epoch for 1 Hz frequency bins ranging from 1–40 Hz. PSD values were then log10 transformed by the software to achieve a Gaussian distribution. Relative PSDs were computed automatically by taking the PSD value in the1 Hz frequency of interest and dividing it by the sum of the PSD values from 1–40 Hz. To obtain bandwidth frequencies, the relative 1 Hz PSD bins were averaged across the frequency ranges as follows: 3–7 Hz (theta), 8–12 Hz (alpha), 13–19 Hz (low beta), 20–29 Hz (high beta), and 30–40 Hz (gamma).

A short-term memory load index (STMLI) was then calculated for each participant and each Nike SST task by dividing relative PSD for theta at Fz by the corresponding relative PSD for gamma, resulting in STMLI ratios for each 1 second epoch (see Formula 1).

$$STMLI\,=\frac{rPSD{\theta }_{Fz}}{rPSD{\gamma }_{Fz}}$$

Formula 1. Calculation of the Short-term Memory Load Index

For each Nike SPARQ test, 1 second epochs of STMLI ratios were aggregated using 5% trimmed means for each participant (i.e., mean STMLI ratios were generated for each SPARQ test for each participant). Five percent trimmed means were used to eliminate 1-second epoch with extreme values.

### Ethical approval

All procedures performed in studies involving human participants were in accordance with the ethical standards of the institutional and/or national research committee and with the 1964 Helsinki declaration and its later amendments or comparable ethical standards.

### Informed consent

Informed consent was obtained from all individual participants included in the study.

## Results

### Nike sensory station measures

The two groups were matched on demographic variables of age (M_mTBI_ = 14.22 vs. M_non-mTBI_ = 13.76, t = −1.18, p=0.24) and sex (χ^2^ = 1.47, p = 0.23). Examination of univariate distributions of scores in both concussion history groups on each of the Nike Sensory Station measures revealed skewed distributions for most of the assessed variables. Additionally, testing for outliers with box plots identified multiple outliers on each of the SST measures. We thus used 5% trimmed means for all of the variables to normalize distributions and minimize heterogeneity of variance between the two groups.

Nike SST measures were then compared between the two groups using independent -sample t-tests with reported t-values based on the between-group Levene’s tests for homogeneity of variance. After applying the Bonferroni correction the results showed that the group with a history of concussion still had significantly poorer depth perception (*M* = 173.13, *SD* = 88.02; *t* = −3.14) and took significantly longer to capture a target (M = 320.59, SD = 164.71; *t* = −3.00) than the group with no prior history of concussion (*M* = 95.35; *SD* = 78.06 and *M* = 238.68, *SD* = 64.59, respectively). Before the correction the mTBI-history group also showed a significantly poorer performance on the perception span task (M = 31.50, SD = 12.56) than those without the history of concussion (M = 38.70, SD = 10.94, *t* = 2.27). The difference was no longer significant following the adjustment. None of the other Nike SST measures were significant between the two groups. These results are summarized in Table 1.

**Table 1:** Means, standard deviations and t-values for Nike SST measures.

Nike SST Measure	No Concussion History (n = 56)	Concussion History (n = 17)	Mean Difference	95% CI of the Difference	t
	M (SD)	M (SD)			
VC (logMAR)	−0.12 (0.11)	−0.06 (0.11)	−0.06	−0.13 – (−0.00)	−2.02
CS (logCS)	1.42 (0.18)	1.48 (0.28)	−0.07	−0.19–0.05	− 1.23
DP (arcsec)	95.35 (78.06)	173.13 (88.02)	−77.78	−127.57 – (−28.00)	−3.14*
NFQ (# correct)	22.08 (3.74)	24.29 (4.88)	−2.21	−4.40 – (−0.02)	−1.73
PS (total score)	38.70 (10.93)	31.50 (12.16)	7.20	0.88–13.52	2.27
HRT(msec)	391.48 (42.55)	387.76 (34.14)	3.72	−19.22–26.66	0.32
EHC (msec)	58,363.26 (4,867.19)	57,983.06 (5,558.73)	380.20	−2,362.25–3,122.66	0.28
TC (msec)	238.68 (64.59)	320.59 (164.71)	−81.91	−136.33 – (−27.49)	−3.00*
GNG (total score)	9.41 (6.36)	12.44 (8.46)	−3.02	−6.85–0.75	−1.58

Note. VC = Visual Clarity, CS = Contrast Sensitivity with 18 cycles per degree, DP = Depth Perception, NEF = Near-far-Quickness, PS = Perception Span, HRT = Hand Response Time, EHC = Eye-Hand Coordination, TC = Target Capture, GNG = Go-no-Go.

*- significant alpha 0.05 after Dunn-Boneferroni adjustment with critical t value = 2.82.

### EEG STMLI ratios

A 2 (Group) × 9 (Test) mixed model ANOVA was used to analyze EEG STMLI ratios. History of concussion served as the between-subjects variable and Test served as the within-subjects variable. We screened for outliers using significant Mahalanobis distances (n = 4). The Greenhouse-Geisser epsilon adjustment was applied as a correction to the degrees of freedom in those analyses, in which a significant Mauchly’s test indicated a violation of the sphericity assumption for the within-subjects factor. The Bonferroni correction was used where appropriate for multiple comparisons.

**Figure 1:**
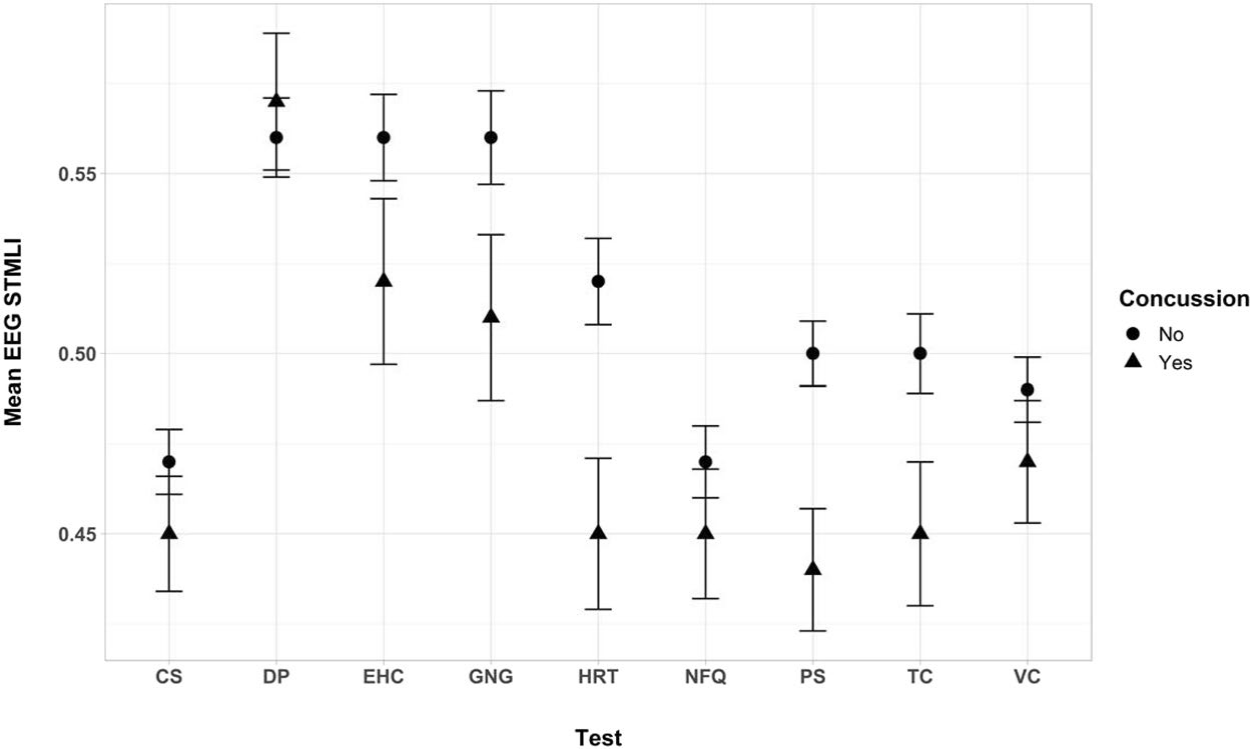
EEG STMLI ratios as a function of Concussion and Test. VC = Visual Clarity, CS = Contrast Sensitivity, DP = Depth Perception, NEF = Near-far-Quickness, PS = Perception Span, HRT = Hand Response Time, EHC = Eye-Hand Coordination, TC = Target Capture, GNG = Go-no-Go.

The 2 × 9 mixed ANOVA revealed a significant main effect for the history of concussion, *F*(1, 71) = 4.24, *p* = 0.043, *η*_p_^2^ = 0.06. Those with a history of concussion (*M* = 0.478, *SE* = 0.015) had significantly lower STMLI ratios across the Tests than those without a history of concussion (*M* = 0.512, *SE* = 0.008). There was also a significant main effect for Test, *F*(5.41, 384.20) = 27.13, *p* < 0.001, *η*_p_^2^ = 0.28. Several significant differences were found between the Tests; however, these main effects were qualified by a significant Concussion × Test interaction, *F*(5.41, 384.20) = 2.40, *p* = 0.033, *η*_p_^2^ = 0.03. Those with a history of concussion had lower STMLI scores during the Perception Span, Hand Response Time, and Target Capture tests (see Table 2). This interaction is also visually depicted in Fig. 1.

**Table 2:** Descriptive Statistics for EEG STMLI ratios by Concussion History and Test.

NIKE SST Test	No Concussion History (*n* = 56)		Concussion History (*n* = 17)		*p*
	*M*	*SE*	*M*	*SE*	
VC	0.49	0.009	0.47	0.017	0.344
CS	0.47	0.009	0.45	0.016	0.262
DP	0.56	0.011	0.57	0.019	0.746
NFQ	0.47	0.010	0.45	0.018	0.435
PS	0.50	0.009	0.44	0.017	**0.007**
HRT	0.52	0.012	0.45	0.021	**0.006**
EHC	0.56	0.012	0.52	0.023	0.119
TC	0.50	0.011	0.45	0.020	**0.023**
GNG	0.56	0.013	0.51	0.023	0.082

Note. VC = Visual Clarity, CS = Contrast Sensitivity, DP = Depth Perception, NEF = Near-far-Quickness, PS = Perception Span, HRT = Hand Response Time, EHC = Eye-Hand Coordination, TC = Target Capture, GNG = Go-no-Go.

The results of the 2 × 9 ANOVA indicated that those with a history of concussion demonstrated different cortical activity during the performance of certain sensory station tests than those without. To reduce the number of station tests down to a more parsimonious number we further conducted a principle components analysis (PCA) and derived component scores for this reduced number of tests to be used as outcome measures.

### PCA on *EEG STMLI ratios*

Initial analysis of the STMLI ratios for the 9 station tests revealed a Kaiser-Meyer-Olkin measure of sampling adequacy of 0.85 (meritorious) and a significant Bartlett’s Sphericity Test, *χ*^2^(36) = 410.64, *p* < 0.001, indicating that that the 9 variables were sufficiently correlated for dimension reduction analysis. The 9 EEG STMLI variables were then subjected to a PCA using varimax rotation to increase solution interpretability. A two-component solution was obtained using a semi-conservative eigenvalue cutoff of 0.80 and investigating a scree plot for an inflection point. Combined, these two components accounted for 70.01% of the variability in the original data. Component loadings are displayed in Table 3. Contrast sensitivity, visual clarity, depth perception, target capture, and near-far quickness loaded on the first component (Component 1). These tests did not carry a significant cognitive processing component. Go-no-go, eye-hand coordination, hand reaction time, and perception span loaded highly onto the second component (Component 2). These tests required more cognitive processing than those included in Component 1. Therefore, the results of the PCA support a common structure among certain tests in terms of STMLI ratios – tests requiring more cognitive processes and psychomotor responses were produced different EEG activation compared to those with less cognitive processing requirement and psychomotor responses.

**Table 3:** Component Loading for PCA on EEG STMLI ratios.

NIKE SST Test	Component 1	Component 2
CS	**0.89**	0.27
VC	**0.86**	0.22
DP	**0.74**	0.32
NFQ	**0.73**	0.40
TC	**0.49**	0.36
GNG	0.28	**0.85**
EHC	0.26	**0.84**
HRT	0.29	**0.76**
PS	0.50	**0.68**

Note. VC = Visual Clarity, CS = Contrast Sensitivity, DP = Depth Perception, NEF = Near-far-Quickness, PS = Perception Span, HRT = Hand Response Time, EHC = Eye-Hand Coordination, TC = Target Capture, GNG = Go-no-Go.

**Figure 2:**
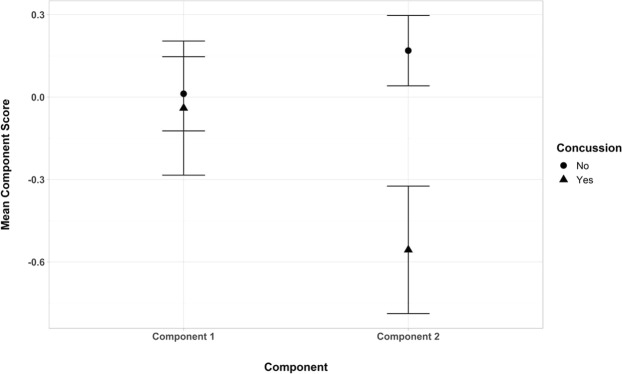
Mean Anderson-Rubin component scores for EEG STMLI ratios by Component and Concussion.

We then computed Anderson-Rubin scores for the two components and subjected these scores to a 2 (History of Concussion) × 2 (Component) mixed model ANOVA, with Component as the within-subjects variable. There was a significant main effect of Concussion history, *F*(1, 71) = 4.10, *p* = 0.047, *η*_p_^2^ = 0.06. Paralleling previous results, those with a history of concussion (*M* = −0.298, *SE* = 0.168) had significantly lower component scores overall than those without (*M* = 0.090, *SE* = 0.093). There was no main effect for Component, *F*(1, 71) = 0.87, *p* = 0.355, *η*_p_^2^ = 0.01. The Concussion × Component interaction approached significance, *F*(1, 71) = 3.04, *p* = 0.086, *η*_p_^2^ = 0.04. Comparing the concussion groups at each level of Component, no differences were found between the concussion groups for tests not requiring more cognitive processing (Component 1). The marginal significance was due to those with a history of concussion (*M* = −0.556, *SE* = 0.232) having more negative Component 2 scores than those without a history of concussion (*M* = 0.169, *SE* = 0.128). Figure 2 displays this trend.

## Discussion

### STMLI ratio and psychomotor ability

Consistent with the previous findings by Mihalik and Wasserman^18^, youth athletes with a history of concussion in our sample had significantly poorer depth perception for the primary gaze than those without the history of concussion. These findings on their own are not particularly surprising, as persistent oculomotor abnormalities including deficits in accommodation, saccadic eye movements, and vergence have been frequently reported in individuals with a history of mTBI (see Ciuffreda *et al*.^37^ for a review).

Significant differences in the EEG ratio of relative frontal PSD of theta to gamma, however, in the present study suggested further deficits in the group of youth athletes with a history of concussion that reach beyond persistent oculomotor abnormalities and may involve working memory decrements in conjunction with processing speeds. Specifically, the theta-to-gamma ratio was significantly lower in the concussion history group on the Nike SST tests of target capture, perception span and hand reaction time.

Our principle component analysis indicated that this EEG metric reflects an underlying dimension that is shared by perception span, hand reaction time, eye hand coordination, and Go/No-Go tests of the Nike battery. Incidentally, behavioral performance on the same exact 4 tests in the study by Wang *et al*.^30^ was shown to load heavily on the underlying dimension of visuo-motor control. In their paper, Chaiken *et al*.^29^ demonstrated that psychomotor performance on visual-motor control tasks largely depends on the working memory capacity and temporal processing speeds. Both working memory and timing ability are thought to predict accuracy and quickness of motor responses^38^. We thus propose that the short-term memory load index reflects efficiency of the psychomotor ability, i.e. a working memory load under temporal processing constraints on visuo-motor tasks, on which either the presentation of stimulus (e.g. perception span) or the response to a stimulus (e.g. eye-hand coordination) have a limited time window.

Indeed, when we reran our analyses using component scores based on STMLI ratios, generally the group of youth athletes with a history of concussion had significantly lower component scores than the group with no concussion history. Moreover, differences for the second component scores (tests of visuo-motor control) showed a greater separation between the two groups that was marginally significant. One of the reasons this difference was not statistically significant may be due to the fact that STMLI mean differences were observed between the groups for target capture. This test, however, was included into the first component, although its loading on the first component was only moderately greater (0.49) than on the second component (0.36). This cross-loading pattern can be explained by a strong temporal processing aspect of the task, as it primarily targets the speed with which the direction of the opening in the Landolt ring is identified.

### Theta-gamma abnormalities in mTBI

Overall, the group of athletes with a history of concussion had significantly lower theta-to-gamma ratios and consequently lower component scores than the athletes with no prior history of concussion. We propose that these lower ratios correspond to greater short-term memory loads. Specifically, lower ratios indicate either lower relative amplitude-based PSDs for frontal theta and/or larger PSDs for frontal gamma in those with a history of concussion. In our study, athletes in the concussion history group had significantly larger relative gamma PSD (*M* = 2.54, *SE* = 0.05) than those with no previous history of concussion (*M* = 2.42, *SE* = 0.02). No significant changes between the groups were observed for the relative PSD of theta at Fz (*M*_*concussed*_ = 1.21; *SE* = 0.03; *M*_*non-concussed*_ = 1.24; *SE* = 0.02). Theoretically, using the power law of an inverse relationship between amplitude and frequency, greater gamma amplitudes would indicate lower relative gamma frequencies in the group of athletes with a history of concussion. Hypothetically, this could have resulted in fewer phase-locked gamma cycles locked within a theta cycle in the group with a history of concussion, which, according to Lisman and Jensen^31^, should delimit working memory capacity and result in poor performance.

There is also some empirical support to this hypothesis. In a series of *in vivo* and *in vitro* recordings Atallah and Scanziani^39^ showed that gamma oscillations in the CA3 region of the rat hippocampus undergo rapid variability in amplitude, and that the amplitude of each oscillation cycle predicts the interval to the next cycle. Specifically, the researchers showed that with larger synaptic currents the duration of the interval to the next gamma cycle was longer, giving rise to frequency variations ranging between 28 to 75 Hz.

Gamma oscillations are generated through a feedback mechanism, in which firing of pyramidal cells and the release of glutamate excites a special population of interneurons, which then rapidly inhibit the entire population of pyramidal cells by releasing GABA^40^. The source of gamma oscillations has been attributed to the activity these GABAergic interneurons. De Beaumont *et al*.^41^ found an increase in GABA-B-mediated inhibition in asymptomatic athletes with at least two concussions at least 9 months following their last injury. The authors suggested a long-term compensatory mechanism against glutamate excitotoxicity to explain their findings. This report adds further evidence to a potential persistent disruption in the theta-gamma coupling as a chronic consequence of mild TBI that in the present study was indexed by lower relative theta-to-gamma amplitude ratios.

The current study was limited in scope with only 18 participants reporting lifetime concussion history, which requires further replication of the current findings in a larger mTBI sample. If our STMLI ratio is indeed related to working memory load under temporal processing constraints, it would be of interest to systematically manipulate each of the constituent variables in a battery of psychomotor tests.

### Conclusions

The results of our study suggest that persistent neuropsychological deficits following concussion may be more subtle than what can be observed with available computerized test batteries. Our electrophysiological index of short-term memory load seems to be a more sensitive measure of mTBI history that in the future may potentially be used in conjunction with both diagnostics and rehabilitation of mTBI. It may be directly applicable to the customized design and implementation of Sports Vision programs aimed at improvement of sports-specific performance. This would be consistent with a trajectory-oriented approach emphasized in a comprehensive review of risks and prognostic factors for concussion outcomes by Collins *et al*.^42^.

Moreover, using this ratio may have implications for other domains outside of sports, in which concussions are common. For example, concussions in military service members are a leading concern for U.S. military operations^43^. EEG theta-gamma ratios combined with visual-motor testing may offer an improved means for identifying concussions in service members experiencing head impacts. Furthermore, theta-gamma ratios may also be used to track return-to-duty progress.

## Data Availability

The datasets generated during and/or analyzed during the current study are not publicly available as this study was part of a larger study and not all data have been yet analyzed and published. Data pertaining to this manuscript are available from the corresponding author on reasonable request.
